# Impact of Telehealth and Social Drivers of Health on Utilization of Fetal Center Services

**DOI:** 10.3390/children13040538

**Published:** 2026-04-13

**Authors:** Nicole M. Khanna, Suhagi Kadakia, Quyen M. Diep, Rakhee M. Bowker

**Affiliations:** 1 Rush Medical College, Rush University Medical Center, Chicago, IL 60612, USA; 2 Division of Neonatology, Department of Pediatrics, Rush University Medical Center, Chicago, IL 60612, USA; 3 Rush Biostatistics Core, Rush University Medical Center, Chicago, IL 60612, USA

**Keywords:** fetal therapy, social drivers of health, area deprivation index, healthcare utilization, congenital anomalies

## Abstract

**Highlights:**

**What are the main findings?**

• Despite the implementation of telehealth to maintain care continuity during the COVID-19 pandemic, our specialized fetal care center experienced a significant decrease in family conference participation compared to the pre-telehealth era.
• Higher neighborhood deprivation predicted higher maternal attendance and lower co-parent attendance at fetal center family complex care coordination conferences in our study.
• Co-parent attendance at family complex care coordination conferences was lower for mothers with Medicaid in our study.

**What are the implications of the main findings?**

• Implementation of telehealth significantly influenced fetal therapy center care utilization patterns.
• Virtual care may improve equity in maternal access to specialized fetal care services, but significant barriers to paternal involvement remain, especially for families with Medicaid or living in neighborhoods with high deprivation.
• The finding of increased depression/anxiety in mothers with low neighborhood deprivation scores compared with mothers residing in more deprived neighborhoods during the COVID-19 era highlights a need for further studies to evaluate the non-linear relationship between socioeconomic status and perinatal mental health.

**Abstract:**

**Background:** Data is limited regarding the impact of social drivers of health (SDOH) on fetal center (FNMC) service utilization. **Objectives:** The objectives of this study were to (1) compare FNMC utilization by era (pre-telehealth era vs. COVID-19 telehealth era) and (2) examine associations between maternal sociodemographics and FNMC service utilization. **Study Design:** A retrospective study of 300 high-risk pregnant individuals with suspected fetal anomalies was conducted, analyzing utilization vs. SDOH (deprivation (ADI), preferred language, race/ethnicity, insurance, employment) comparing pre-telehealth (n = 200) and COVID-19 telehealth (n = 100) cohorts. **Results:** Race/ethnicity was associated with differences in fetal echo utilization and family complex care coordination conference (FC) attendance in the univariate analysis. The ADI decile and era predicted FC attendance in the logistic regression, while race/ethnicity, preferred language, and employment were not significant. The ADI and insurance were significant predictors of co-parent attendance, whereas era, employment, preferred language, and race were not. **Conclusions:** FC attendance was lower in the COVID-19 era despite telehealth expansion. Higher neighborhood deprivation predicted higher maternal attendance but lower co-parent attendance. Co-parent FC attendance was lower for mothers with Medicaid.

## 1. Introduction

Birth defects, otherwise known as congenital anomalies, represent the leading cause of infant mortality in the United States as of 2026, accounting for 1 in 5 (20%) of all infant deaths [1]. Congenital anomalies affect 3–4 percent of live births (150,000 infants) annually in the United States, and 25,000 neonates are born in the U.S. each year with multiple anomalies [2,3]. These may include central nervous system malformations, cleft lip and palate, congenital heart disease, lung malformations, abdominal wall defects, genitourinary tract anomalies, and genetic syndromes. Newborns with congenital anomalies often require specialized care in the neonatal intensive care unit (NICU) after birth. These complex newborns require coordination of complex care, sometimes including consultation by multiple pediatric subspecialists, and may require surgical interventions in the immediate newborn period in order to survive and thrive. Accordingly, maternal–fetal dyads affected by congenital anomalies during pregnancy benefit from specialized fetal care to optimize outcomes [4,5].

Fetal therapy centers have emerged to address this complexity by integrating expertise across maternal–fetal medicine, neonatology, pediatric surgery, genetics, and other subspecialties. These centers provide comprehensive prenatal diagnosis, multidisciplinary counseling, and coordinated care planning. Central to this process are fetal complex care coordination conferences (FCs), which facilitate shared decision making and allow families to engage with multiple providers simultaneously to understand risks, benefits, and expected outcomes, as per ACOG guidelines [6]. Studies have shown that families affected by congenital anomalies who receive care at fetal centers report higher satisfaction [7]. Multidisciplinary prenatal counseling for fetal surgical anomalies has been shown to reduce maternal anxiety compared to obstetrician-only counseling [8,9]. For fetal therapeutic interventions, such as fetoscopic tracheal occlusion for congenital diaphragmatic hernia [10,11] or catheter-based cardiac procedures [12], time-sensitive decisions are made in consultation with the team to optimize fetal and maternal outcomes.

One of the major adjustments to healthcare practices during the Coronavirus Infectious Disease-19 (COVID-19) pandemic was the rapid adoption of telemedicine to mitigate disease spread. Prior to 2020, telemedicine was used to provide highly specialized care in geographical areas where patients would have to drive long distances for in-person visits [13,14]. Due to the COVID-19 pandemic, the Center for Medicaid and Medicare Services (CMS) changed the coding and billing codes for telehealth services to allow telehealth services to be reimbursed by many payors as face-to-face services to encourage providers to transition to telemedicine to help mitigate disease spread [15]. The widespread adoption of telehealth across disciplines included fetal therapy centers and MFM providers who provide prenatal care for high-risk patients. Virtual telehealth visits did not fully replace in-person encounters during prenatal care but offered a means of reducing potential exposures of patients and providers to COVID-19 and facilitated consolidated in-person testing and services [16].

Hargis-Villanueva and colleagues at Phoenix Children’s Hospital assessed provider satisfaction with the rapid transition from in-person prenatal visits to multidisciplinary consultations via telehealth as an adaptive response to the pandemic. The providers surveyed highlighted convenience and improved care coordination across specialties [17]. Virtual telehealth visits enhance patient care by increasing accessibility through flexible scheduling and the elimination of geographic and travel barriers [18]. Furthermore, this modality reduces childcare expenditures and minimizes the risk of infectious disease transmission across diverse clinical environments. Telemedicine also facilitates care within the home environment and, during the pandemic, enabled greater familial support by circumventing clinical visitor restrictions. However, virtual visits also have limitations, including reliance on internet access, communication challenges for patients with language barriers, limited physical exams, and potential concerns regarding patient privacy depending on the location of the telehealth encounter. Barriers to digital literacy may be more pronounced for patients living in deprived neighborhoods, based on access to technology (mobile phones, tablets, laptops) and reliable internet access, as well as knowledge of how to navigate patient portals. The impact of telehealth on improving access to specialized fetal care for families whose pregnancies are affected by congenital anomalies or high-risk maternal conditions and the ways that social drivers of health (SDOH) may influence healthcare utilization in the era of telehealth are currently unknown.

In the United States, Black women experience adverse maternal health outcomes, with maternal morbidity and mortality ratios several times higher than those of other groups [19,20]. SDOH, including neighborhood deprivation, race/ethnicity, and insurance status, are known to influence healthcare access and outcomes [21]. The Area Deprivation Index (ADI), a composite measure of neighborhood-level disadvantage, has been associated with adverse perinatal outcomes, including preterm birth [22]. However, the relationship between the ADI and utilization of specialized fetal care services, particularly in the context of telehealth, is not well understood. Giron and colleagues evaluated the relationship between geography and socioeconomic status and access to specialized fetal care for congenital anomalies in the United States and found that over 40% of women of child-bearing age resided in “fetal care deserts,” defined as ZIP codes ≥100 miles away from the nearest North American Fetal Therapy Network (NAFTNet) center [23]. They also looked at the ADI using ZIP code-level data from the American Community Survey and determined that in the 40% of women of child-bearing age that lived ≥ 100 miles from a NAFTNet center, the median income was significantly lower, and the rates of poverty, unemployment, uninsured status, and the ADI were significantly higher. The authors proposed that the targeted expansion of fetal therapy centers may address disparities and improve access to care.

To address these disparities, telemedicine could be a vital tool for improving care access for patients living in fetal care deserts. While geographic distance from a fetal therapy center to access specialized fetal care is a primary barrier, healthcare utilization also may be limited by medical mistrust, language discordance, and other unidentified social factors. Thus, further study of healthcare utilization patterns by maternal–infant dyads affected by congenital anomalies is needed to highlight potential barriers to accessing the highly specialized tertiary or quaternary care that can be lifesaving for this uniquely vulnerable patient population.

The primary objective of this study is to compare fetal center service utilization by high-risk pregnant individuals affected by congenital anomalies between the pre-telehealth and telehealth eras. The secondary objectives include examination of the association between SDOH (neighborhood deprivation, maternal preferred language, maternal race/ethnicity, maternal insurance, maternal employment) and fetal center service utilization, as well as exploration of the support networks for mothers (co-parent FC attendance and FC attendance by other supporting adults). We hypothesized that telehealth would modify the association between the ADI and healthcare utilization. Specifically, we hypothesized that hosting FCs via telehealth encounters would improve utilization by mitigating barriers to attendance for mothers living in highly deprived areas as measured by ADI deciles. We further hypothesized that telehealth would increase the presence of co-parents (or other supporting adults) at FCs in the telehealth era by permitting remote attendance.

## 2. Materials and Methods

### 2.1. Study Population and Design

This was a retrospective study of a convenience sample of pregnant mothers with fetal anomalies, genetic diagnoses or high-risk maternal conditions who were referred to an urban academic fetal therapy center between January 2014 and December 2019 (pre-telehealth era) and between June 2020 and May 2023 (COVID-19 telehealth era).

The Rush Fetal and Neonatal Medicine Center (FNMC) is a level 3 fetal therapy center [4] within an urban academic medical center that provides multidisciplinary care for pregnancies complicated by fetal anomalies or high-risk maternal conditions requiring specialized newborn care, such as abnormal placentation, multiple gestations, or maternal oncologic diagnoses. Complex cases benefit from coordinated care from maternal–fetal medicine (MFM), neonatology, and pediatric medical and surgical subspecialists to ensure optimal outcomes for both mother and baby in tertiary or quaternary care settings. Pregnant individuals referred to the FNMC are invited to participate in a multidisciplinary family complex care FC to plan for the medical needs of their infant during pregnancy, at delivery, and in the NICU or nursery after birth. Based on the congenital anomaly suspected or high-risk maternal condition, fetal center patients are scheduled for in-person MFM consultations and advanced imaging, including ultrasound, echocardiogram, and/or fetal MRI. All FNMC patients with suspected congenital anomalies are also referred for genetic counseling. Genetic counseling transitioned to the telehealth platform during the pandemic.

Prior to 2020 and the COVID-19 pandemic, multidisciplinary FCs were held in person at the medical center on an outpatient basis in the FNMC, with no virtual telehealth option. During the pandemic, the Rush FNMC transitioned to a virtual platform for multidisciplinary meetings, and all outpatient FCs were held via video telehealth visits. At the onset of the pandemic, these video visits were held via a secure video platform, Zoom^®^ versions 5 and 5.3.0 (Zoom Video Communications, Inc., San Jose, CA, USA, 2020). Zoom is accessible via desktop browser or by mobile phone application. On a desktop or laptop computer, users require a microphone and speaker at minimum, and a webcam allows the user to show themselves on video. Mobile smart phones typically have built-in microphones and cameras. For patients who did not have access to the technology required or had difficulty navigating the technology, the fetal center RN coordinator called the patient at the phone number they provided and allowed the patient to attend the conference by phone, with the remaining multidisciplinary provider team on the video platform. Interpreter services joined family conferences for patients whose preferred language was not English. The video visit platform transitioned from Zoom to Epic^®^ (Epic Version November 2022 by Epic Systems Corporation, Verona, WI, USA) MyChart electronic health record-based telehealth visits by the end of the pandemic.

The COVID-19 emergency declaration officially ended on 11 May 2023, and therefore patients referred to the fetal center after 1 May 2023 were excluded from this study since the Rush FNMC was then able to offer both in-person and virtual visits for outpatient FCs [24]. Subjects were identified from the FNMC Database of Maternal and Infant Data, which is used internally for quality improvement (QI) and budgeting purposes. Inclusion criteria were as follows: pregnant individuals referred to the Rush FNMC between 1 January 2014 and 31 December 2019 (pre-telehealth era) or between 1 June 2020 and 1 May 2023 (COVID-19 telehealth era) who qualified for a multidisciplinary fetal center complex care coordination conference based on the presence of a suspected fetal congenital anomaly, suspected genetic diagnosis or syndrome, or high-risk pregnancy requiring special care or considerations for the delivering parent and/or baby during or after delivery. Two-hundred pre-telehealth-era mother–infant dyads and 100 COVID-19 telehealth-era mother–infant dyads met these inclusion criteria and were included in this study. The Rush IRB approved this study, and informed consent was waived.

### 2.2. Data Abstraction

Chart reviews were performed, and the following data were abstracted from the medical record: FNMC intake date, including month and year; mother’s age and parity at time of FNMC referral; maternal race and ethnicity; maternal address and zip code; maternal preferred language and use of interpreter; maternal primary insurance; maternal employment status; maternal reported level of education completed; maternal comorbid diagnoses; suspected fetal diagnosis. Fetal center service utilization data during the current pregnancy were also abstracted from the medical record, including: MFM consultation; fetal imaging performed (fetal MRI, fetal echocardiography, fetal ultrasound); antenatal genetic counseling acceptance/attendance; whether an FC was offered, accepted, and attended by the pregnant individual, as well as FC attendance by other supporting adults and their relationship to the pregnant patient as described in the medical record; and whether a co-parent was reported by the pregnant individual as involved in the pregnancy.

### 2.3. Statistical Analyses

Analysis of de-identified data was performed with SPSS^®^ Statistics Software version 22 (Chicago, IL, USA) and Microsoft Excel. In this study, we utilized the mother’s Area Deprivation Index (ADI) decile as the variable representing the mothers’ neighborhood deprivation. The mothers’ ADI deciles were calculated using an interactive map that publishes state ADI deciles online using 2018 United States (U.S.) Census Bureau data [25,26]. The ADI is a metric that measures area disadvantage, determined by 17 census variables, including measures of poverty, educational level, housing, and employment status, described in relation to disparities in life expectancy that correlate with area deprivation. The ADI has been adapted, updated, and validated at the neighborhood level using block groups, the smallest geographical unit used by the U.S. Census Bureau, and it has been normalized at the national level to generate percentiles with higher values indicating greater disadvantage. Of note, one patient’s score from the pre-telehealth era could not be reported due to an indeterminate value for the block group. A *p*-value < 0.05 was considered statistically significant.

Univariate analysis included *t*-tests, Mann–Whitney U tests, and chi-square tests based on the appropriate distribution of variables. A binary logistic regression analysis was performed evaluating mothers’ FC attendance in a model which included the era (pre-telehealth era vs. COVID-19 telehealth era), maternal ADI decile, as well as maternal preferred language, maternal insurance, maternal employment status, and maternal race/ethnicity. Binary logistic regression analysis was also performed evaluating father/co-parent attendance at the FC. Fetal service utilization (fetal echo, fetal ultrasound, fetal MRI, MFM consultation, genetic counseling, and FC meeting) was also analyzed by ANOVA stratified by maternal preferred language and maternal race/ethnicity.

## 3. Results

A total of 300 high-risk pregnant mothers with suspected congenital anomalies were identified within the study period, with 200 from the pre-telehealth-era cohort and 100 from the COVID-19 telehealth-era cohort. Maternal sociodemographic characteristics stratified by era (pre-telehealth vs. telehealth) are reported in Table 1. Maternal sociodemographic characteristics stratified by low-ADI-decile (1–3, low neighborhood deprivation) and high-ADI-decile (7–10, high neighborhood deprivation) groups are reported in Table 2. Utilization of FNMC services stratified by era (pre-telehealth vs. telehealth cohorts) is reported in Table 3. Utilization of FNMC services stratified by low- and high-ADI-decile groups is reported in Table 4.

FC utilization by pregnant mothers referred to the FNMC with suspected fetal anomalies or high-risk maternal conditions decreased during the COVID-19 telehealth era, despite the transition to telehealth video visits (Table 3). FC attendance was significantly higher in pre-telehealth-era mothers when compared with COVID-19 telehealth-era mothers (84% of pre-COVID-19-era mothers attended FCs vs. 67% of COVID-19-era mothers, *p* = 0.002). FC meetings were offered to significantly fewer mothers with pregnancy complicated by suspected fetal anomalies during the COVID-19 telehealth era (91% pre-telehealth era vs. 77% COVID-19 telehealth era, *p* = 0.006). For those mothers who accepted the offer of attending an FC meeting, the numbers and compositions of support persons differed significantly, with increased participation by supporting adults in the pre-telehealth era, including increased attendance by fathers or co-parents (69% pre-telehealth era vs. 54% COVID-19 era, *p* = 0.039). The composition of supporting adults also differed significantly, as shown in Table 3 (*p* = 0.005), with 39% of COVID-19 telehealth-era mothers attending the FC meeting alone, compared with 19% of mothers in the pre-telehealth-era cohort. Multiple support persons accompanied six percent of COVID-19-era mothers to the FC, as compared with 18% in the pre-telehealth-era cohort (Table 3). Fathers (or co-parents) attended FC meetings significantly more often in the pre-telehealth era (69% vs. 54%, *p* = 0.039).

When stratified by high and low ADI deciles, mothers living in highly deprived neighborhoods showed no difference in fetal imaging utilization, MFM consultation, or genetic counseling visits in the univariate analysis. Comparing mothers with high vs. low ADI deciles, we found differences in the total numbers of FC participants (2.07 participants for low-ADI mothers vs. 1.88 participants for high-ADI mothers, *p* = 0.039) and the compositions of supporting adults at their FC meetings (Table 4) based on the neighborhood deprivation of the mothers. Mothers living in highly deprived neighborhoods attended more FC meetings alone with no support person compared with low-ADI mothers living in more affluent neighborhoods (36% vs. 15%, *p* = 0.012) and had decreased co-parent/father FC attendance compared with low-ADI mothers (52% vs. 75%, *p* = 0.003), as depicted in Table 4.

A binary logistic regression analysis was performed evaluating mothers’ FC attendance in a model which included era (pre-telehealth era vs. COVID-19 telehealth era), maternal preferred language, maternal insurance, maternal employment status, maternal ADI deciles, and maternal race/ethnicity. The full model containing all predictors was statistically significant (*X*^2^ (N = 300) = 42.1, *p* < 0.001). The model explained between 13.2% (Cox & Snell *R*^2^) and 20.2% (Nagelkerke *R*^2^) of the variance in FC attendance. The era was a significant predictor of FC utilization (*B* = −0.88, *p* = 0.006); mothers in the COVID-19 telehealth-era cohort had lower odds of attending an FC compared to pre-telehealth-era mothers (OR = 0.42). Maternal race/ethnicity, maternal preferred language, and maternal employment status did not significantly correlate with the mothers’ FC attendance. The ADI decile was a significant predictor of FC attendance (*B* = 0.19, *p* = 0.006): as the ADI decile increased, the OR of FC attendance increased by 1.2.

Co-parent attendance at the family complex care coordination conferences was also analyzed via a binary logistic regression model. The full model containing all predictors was statistically significant (*X*^2^ (N = 300) = 38.9, *p* < 0.001). The model explained between 15.4% (Cox & Snell *R*^2^) and 21.1% (Nagelkerke *R*^2^) of the variance in the co-parent FC attendance. In the regression analysis, era (COVID-19 telehealth era vs. pre-telehealth era) was not a significant predictor of co-parent FC attendance. We found that maternal insurance was a significant predictor of co-parent attendance at FCs (*B =* −0.93, *p* = 0.019); mothers with Medicaid had lower odds of the co-parent attending the FC compared with mothers with private insurance (OR = 0.40). Additionally, we found that the maternal ADI decile was a significant predictor of co-parent FC attendance (*B* = −0.14, *p* = 0.05): for every decile increase in the ADI (meaning higher area deprivation risk), the odds of a co-parent attending an FC meeting decreased by 0.87. Maternal employment status, maternal preferred language, and maternal race were not significant predictors of co-parent FC attendance in the regression analysis.

**Figure 1:**
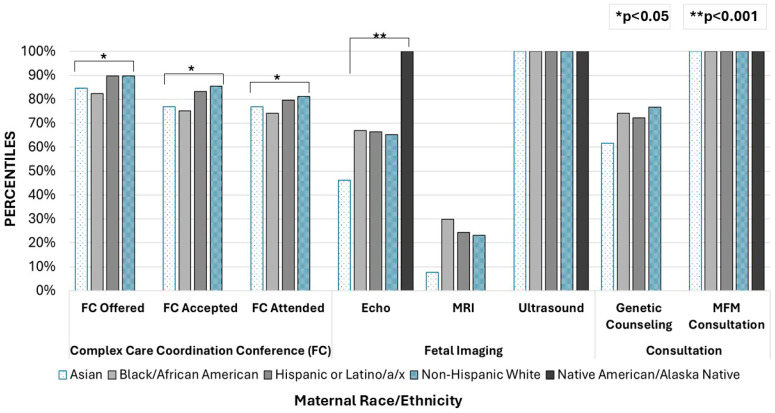
Fetal center service utilization % by maternal race/ethnicity. When fetal center service utilization was analyzed stratified by maternal race/ethnicity via ANOVA, results were notable for significant differences in fetal echo utilization (*X*^2^ = 13.7, *p* < 0.001), FC offered (*X*^2^ = 7.32, *p* = 0.026), FC accepted (*X*^2^ = 5.82, *p* = 0.16), and FC attended (*X*^2^ = 6.3, *p* = 0.012). There were no significant differences in MRI utilization, US utilization, MFM consultation or genetic counseling attendance when evaluated by maternal race and ethnicity.

When fetal center service utilization (including echo, fetal US, fetal MRI, MFM consultation, genetic counseling, and FC attendance) was analyzed based on maternal preferred language, there were no significant differences found. Fetal center service utilization was also analyzed by maternal preferred language stratified by era (COVID-19 telehealth era vs. pre-COVID-19 telehealth era) and showed no significant differences in fetal therapy healthcare utilization among English speakers, Spanish speakers, and mothers who reported a preferred language other than English or Spanish. Figure 1 depicts fetal center service utilization by maternal race/ethnicity.

**Figure 2:**
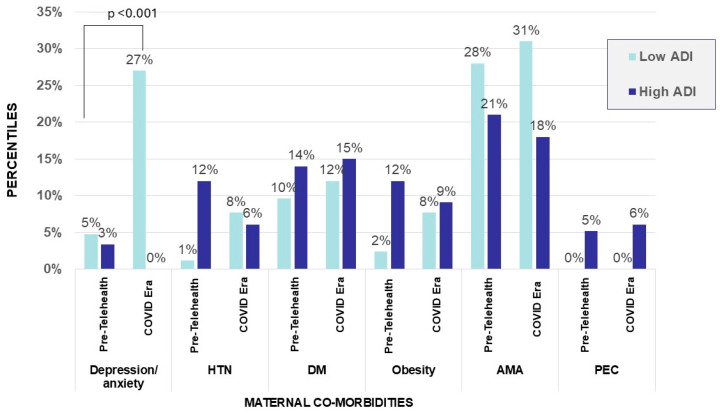
Maternal comorbidities in pre-telehealth era and during COVID-19 telehealth era stratified by ADIs. In COVID-19 telehealth-era cohort, maternal anxiety/depression was significantly higher in low-ADI-decile mothers when compared with high-ADI-decile mothers (27% vs. 0%, *p* < 0.001). Other maternal comorbidities (obesity, pre-eclampsia, hypertension, diabetes, advanced maternal age) did not show significant difference in low- vs. high-ADI-decile mothers when comparing eras (Figure 2).

Evaluation of maternal comorbid diagnoses showed that maternal anxiety/depression increased during the COVID-19 telehealth era (16% vs. 4%, *p* < 0.001), while other maternal comorbid diagnoses, including advanced maternal age, obesity, pre-eclampsia, hypertension, and diabetes, remained unchanged (Table 1). Maternal anxiety/depression was not significantly different in low- and high-ADI-decile mothers in the pre telehealth era (5% vs. 3%, NS), as depicted in Figure 2.

**Figure 3:**
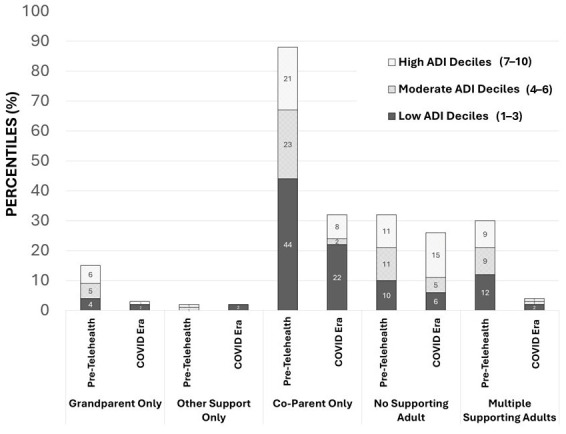
FC supporting-adult attendance in pre-telehealth era compared with COVID-19 telehealth era stratified by ADI deciles. High-ADI mothers had decreased FC supporting-adult attendance during COVID-19 telehealth era compared to low-ADI mothers (*p* = 0.018). During pre-telehealth era, there was no significant difference in FC supporting-adult attendance across ADI deciles in univariate analysis.

Figure 3 depicts the FC supporting-adult attendance in the pre-telehealth-era cohort compared to the COVID-19-era cohort, stratified by low (1–3), moderate (4–6), and high (7–10) ADI deciles. The results showed that high-ADI-decile COVID-19-era cohort mothers had decreased FC supporting-adult attendance compared to low-ADI mothers (*p* = 0.018). In the pre-telehealth era, there were no significant differences in the FC supporting-adult attendance across ADI deciles (Figure 3).

## 4. Discussion

In our retrospective study, we evaluated fetal therapy center service utilization across the pre-telehealth and COVID-19 telehealth eras and examined associations with social drivers of health. We found that telehealth implementation was associated with decreased maternal participation in FCs, despite unchanged utilization of core clinical services. In contrast, era was not a significant predictor of co-parent attendance. We also found that increasing neighborhood deprivation (ADI) was associated with higher maternal FC attendance but lower co-parent participation, and that Medicaid insurance was associated with decreased co-parent attendance. Together, these findings highlight important differences between maternal and family-level engagement in multidisciplinary fetal care.

The existing literature establishes that pregnant individuals in high-ADI neighborhoods face elevated risks for adverse outcomes, including extremely and very preterm births [22]. While neighborhood deprivation is associated with higher rates of impaired glucose tolerance and early-pregnancy hyperglycemia—often driven by structural stressors like food insecurity, psychosocial stress, and higher pre-pregnancy BMI [27,28]—its specific relationship with congenital anomalies remains under-investigated. Although elevated hemoglobin A1c during organogenesis is a known teratogen [27,29], our cohort of high-risk pregnancies with congenital anomalies showed no significant differences in gestational diabetes or other maternal comorbidities across ADI deciles. This likely reflects the high baseline prevalence of diabetes in this specific population. Notably, however, maternal anxiety and depression during the COVID-19 telehealth era did vary significantly by ADI.

In our study, we found decreased odds of maternal FC attendance during the pandemic compared with the pre-telehealth era. We did not identify differences in the co-parent FC attendance based on era. The COVID-19 pandemic acted as a universal stressor that bypassed typical socioeconomic protections for many families. The pandemic exacerbated existing socioeconomic inequalities, leading to heightened anxiety and depression driven by economic instability, resource scarcity, and environmental stressors [30]. We also found increased maternal depression/anxiety in the low-ADI mothers compared with high-ADI mothers during the COVID-19 telehealth era, which may have functioned as a mediator for decreased FC attendance in the low-ADI mothers. The relationship between the ADI and maternal mental health is complex, often reflecting a U-shaped or bifurcated risk profile where both high-deprivation and low-deprivation cohorts experience significant psychological distress [28]. During the pandemic, high-ADI pregnant mothers experienced severe disruptions in income, food insecurity, and housing instability, lack of access to reliable childcare, and environmental stressors, including overcrowding, which made social distancing difficult. While high-ADI mothers struggled with survival-based stressors, low-ADI mothers during the pandemic were uniquely vulnerable to achievement-based stressors and the collapse of the social structures they relied upon to balance their professional and maternal identities [28,31]. Cameron and colleagues also found that mothers in low-deprivation areas reported unexpectedly high levels of anxiety and depression during the pandemic, mirroring our study findings [32]. They found that complex social networks were abruptly severed during pandemic-associated lockdowns, leading to profound isolation for many mothers, as well as an increase in domestic responsibilities fueled by the abrupt loss of outsourced support (nannies, house cleaners, private childcare) [32].

We also noted increased odds of maternal FC attendance with increasing neighborhood deprivation. This finding may have been mediated partially by increased maternal anxiety/depression in low-ADI mothers during the pandemic and by telehealth mitigating logistical barriers to FC utilization for high-ADI mothers. An extensive body of literature published post-pandemic has shown that telehealth mitigates structural and logistical barriers related to transportation, childcare and inflexible work schedules for pregnant individuals living in highly deprived neighborhoods [16,33,34,35]. Virtual visits have been shown to reduce the indirect costs of care and potentially may improve adherence to the intensive monitoring schedules required for anomalous pregnancies. For mothers who reside in maternity care deserts, telehealth has been shown to facilitate real-time consultation with MFM specialists and fetal cardiologists without the burden of long-distance travel. Advanced tele-ultrasound enables specialists at tertiary care centers to remotely interpret imaging conducted at local clinics. This technology facilitates the timely detection of fetal or maternal anomalies for mothers residing in high-ADI regions and may effectively mitigate some of the geographic, logistical, and economic barriers that often preclude in-person consultations.

Our analysis of SDOH further highlights differences in engagement across populations. While a higher ADI was associated with increased maternal attendance at an FC, it was also associated with decreased co-parent participation, indicating that maternal engagement does not necessarily reflect family-level engagement. In addition, Medicaid insurance was associated with lower co-parent attendance. These findings suggest that structural and socioeconomic factors may differentially impact participation among members of the support network. Notably, decreased supporting-adult participation among high-ADI families was observed in the telehealth era but not prior to the pandemic, suggesting that telehealth may exacerbate existing disparities in family engagement.

In the literature, a high ADI is frequently linked to a lack of father-inclusive support in healthcare services and workplaces [36]. This can be exacerbated by financial instability, which forces fathers to prioritize work attendance over attending medical appointments or participating in NICU caregiver education. There have been several studies evaluating the impact of fathers’ participation in prenatal care for pregnancies complicated by congenital anomalies [36,37,38]. Pridham et al. found that paternal involvement in prenatal care—namely, attending appointments and sharing information—strongly predicts long-term engagement in health decisions for children with congenital anomalies [36]. Future study is warranted to evaluate barriers to paternal participation in prenatal care for complex pregnancies.

In our cohort, we found that maternal residence in highly deprived neighborhoods was associated with a decrease in the presence of supporting adults during multidisciplinary fetal care conferences. Both the number of FC participants and the composition of supporting adults at their FC meetings varied significantly based on neighborhood deprivation. Qualitative studies show that extended family support serves as a critical driver of maternal resilience, facilitating both psychological acceptance and practical caregiving for children with congenital anomalies [39]. The presence of supportive maternal grandmothers has been shown to significantly influence the well-being of pregnant and postpartum mothers and can serve as a protective factor against risk of mental health disorders during the perinatal period [40]. The American College of Obstetricians and Gynecologists emphasizes that postpartum care should be an ongoing process including a village of support that includes family members and community resources to ensure healthy transitions [41].

The importance of family and community support in this population is well established. Prior studies have demonstrated that structured prenatal support and peer engagement can reduce isolation, improve coping, and enhance parental preparedness for the care of medically complex infants. In our study, decreased participation of support persons during FCs, along with increased maternal anxiety and depression in certain subgroups, underscores the need to ensure that care models facilitate not only access but also meaningful engagement and support for families.

Our study had several limitations that may limit its generalizability beyond our single center. First, as a retrospective analysis, the findings are subject to inherent biases, including potential inaccuracies in electronic health record documentation and the inability to establish definitive causality between socioeconomic factors and service utilization. Second, while the use of the ADI provides a robust neighborhood-level measure of disadvantage, it does not fully capture individual-level socioeconomic nuances or the subjective psychological experiences of patients. Furthermore, while the COVID-19 pandemic provided a unique natural experiment for telehealth implementation, it also introduced confounding universal stressors—such as social isolation and economic volatility—that may have influenced maternal health and attendance patterns in ways distinct from non-pandemic conditions. In our study, the measurement of co-parent involvement or participation was limited by the electronic health record and may have underestimated paternal support. Additional barriers to father-inclusive support in clinical settings were not studied. Finally, while we presumed that telehealth would mitigate barriers to healthcare utilization, we did not measure digital literacy, availability of technology (mobile devices/tablets/computers) or other barriers to telehealth utilization. Prospective, multicenter qualitative research is likely needed to identify and further explore which factors are the most important to fetal therapy patients and their families as they navigate high-risk pregnancies and anticipate the births of infants with complex medical needs.

## 5. Conclusions

Our findings demonstrate that the implementation of telehealth significantly influenced fetal care utilization patterns. While neighborhood deprivation was associated with increased maternal attendance—likely due to telehealth mitigating logistical and structural barriers—it conversely predicted lower odds of co-parent participation. This suggests that while virtual care may improve equity in maternal access, significant barriers to paternal involvement remain, particularly for families with Medicaid or those facing high levels of neighborhood-level disadvantage. Additionally, the unexpected finding of heightened psychological distress among low-deprivation mothers during the pandemic underscores the complex, non-linear relationship between socioeconomic status and perinatal mental health. Future studies should focus on sustaining the benefits of telehealth for vulnerable populations while developing targeted, inclusive strategies to bolster co-parent support and address the diverse psychological stressors affecting pregnant individuals across the socioeconomic spectrum.

## Figures and Tables

**Table 1:** Maternal characteristics stratified by pre-telehealth and COVID-19 telehealth eras.

Cohort Characteristics ^#^	Pre-Telehealth Era (n = 200)	COVID-19 Telehealth Era (n = 100)	*p* Value
Maternal Age (years)	29.3 ± 6.5	29.9 ± 6.8	NS
Maternal Race/Ethnicity			
*Non-Hispanic White*	51 (26%)	18 (19%)	
*Non-Hispanic Black*	64 (33%)	33 (35%)	
*Hispanic or Latino*	69 (36%)	39 (41%)	NS
*Native American or Alaska Native*	2 (1%)	0 (0%)	
*Asian*	8 (4.1%)	5 (5%)	
*Not reported*	6 (3%)	5 (5%)	
Maternal Preferred Language *			
*English*	164 (82%)	92 (92%)	
*Spanish*	25 (13%)	8 (8%)	0. 03
*Other*	10 (5%)	None (0%)	
*Not reported*	1 (<1%)	None (0%)	
Maternal Insurance *			
*Private or commercial*	83 (42%)	32 (32%)	
*Medicaid*	86 (43%)	65 (65%)	0.015
*Not reported*	31 (16%)	3 (3%)	
Maternal Parity			
*Primiparous*	73 (37%)	44 (45%)	
*Multiparous*	126 (63%)	53 (55%)	NS
*Not reported*	1 (<1%)	3 (3%)	
Maternal High-Risk ADI (7–10)	24 (32%)	24 (37.5%)	NS
Mean ADI Score	5.1 ± 2.4	5.2 ± 2.7	NS
Maternal Comorbid Diagnoses			
*Advanced maternal age*	46 (23%)	23 (23%)	NS
*Obesity*	14 (7%)	7 (7%)	NS
*Pre-eclampsia*	5 (3%)	2 (2%)	NS
*Hypertension*	13 (6.5%)	8 (8%)	NS
*Diabetes*	22 (11%)	12 (12%)	NS
Anxiety/depression *	8 (4%)	16 (16%)	<0.001
Maternal Employment Status			
*Not employed*	113 (57%)	46 (46%)	
*Employed part time*	11 (6%)	5 (5%)	
*Employed full time*	72 (36%)	44 (44%)	NS
*Student*	2 (1%)	1 (1%)	
*Not reported*	2 (1%)	4 (4%)	

Abbreviations: ADI, area deprivation index; NS, not significant; ^#^ data presented as n (%), mean ± SD. * *p* < 0.05.

**Table 2:** Maternal characteristics stratified by low and high ADI deciles.

Cohort Characteristics ^#^	Low ADI Deciles (N = 135)	High ADI Deciles (N = 91)	*p* Value
Cohort/Era			
*Pre-telehealth*	83 (61%)	58 (64%)	NS
*COVID-19 telehealth*	52 (39%)	33 (36%)	
Maternal Age (years) *	30.7 ± 6.4 (6%)	28.9 ± 6.8 (7%)	0.034
Maternal Race/Ethnicity			
*Non-Hispanic White*	48 (38%)	6 (7%)	
*Non-Hispanic Black*	24 (19%)	45 (51%)	
*Hispanic or Latino*	46 (36%)	34 (39%)	<0.001
*Native American/Alaska Native*	0 (0%)	1 (1%)	
*Asian*	10 (8%)	2 (2%)	
*Not reported*	7 (5%)	3 (3%)	
Maternal Preferred Language *			
*English*	121 (90%)	74 (81%)	
*Spanish*	7 (5%)	14 (15%)	0.035
*Other*	6 (5%)	3 (3%)	
*Not reported*	1 (1%)	0 (0%)	
Maternal Insurance *			
*Private or commercial*	73 (59%)	20 (25%)	
*Medicaid*	51 (41%)	59 (75%)	<0.001
*Not reported*	11 (8%)	12 (13%)	
Maternal Parity			
*Primiparous*	61 (46%)	25 (28%)	
*Multiparous*	73 (54%)	64 (72%)	0.013
*Not reported*	1 (1%)	2 (2%)	
Maternal Comorbid Diagnoses			
*Advanced Maternal Age*	39 (29%)	18 (20%)	NS
*Obesity*	6 (4%)	10 (11%)	NS
*Pre-eclampsia*	0 (0%)	5 (6%)	0.022
*Hypertension*	5 (4%)	9 (10%)	NS
*Diabetes*	14 (10%)	13 (14%)	NS
Anxiety/depression *	18 (13%)	2 (2%)	0.008
Maternal Employment Status			
*Not employed*	60 (45%)	55 (62%)	
*Employed part time*	7 (5%)	4 (5%)	
*Employed full time*	63 (48%)	30 (34%)	0.085 (NS)
*Student*	2 (2%)	0 (0%)	
*Not reported*	3 (2%)	2 (2%)	

Abbreviations: ADI, area deprivation index; NS, not significant; ^#^ data presented as n (%), mean ± SD. * *p* < 0.05.

**Table 3:** Utilization of fetal–neonatal center services stratified by era.

Cohort Characteristics ^#^	Pre-Telehealth Era (n = 200)	COVID-19 Telehealth Era (n = 100)	*p* Value
MFM Consultation	200 (100%)	100 (100%)	NS
Fetal Imaging			
*Level 2 ultrasound*	199 (99.5%)	100 (100%)	NS
*Fetal MRI*	56 (28%)	22(22%)	NS
Fetal echo *	143 (72%)	53 (53%)	0.002
Genetic Counseling Visit	144 (72%)	76 (76%)	NS
FC Meeting Utilization			
Offered *	182 (91%)	77 (77%)	0.006
Accepted *	172 (86%)	70 (70%)	0.002
Attended *	167 (84%)	67 (67%)	0.002
FC Participants			
Total number attending *	2.11 ± 0.90	1.76 ± 0.85	<0.001
FC Supporting-Adult Composition *			
*No support person*	32 (19%)	26 (39%)	
*Co-parent or partner only*	88 (52%)	32 (48%)	
*Grandparent only*	15 (8.9%)	3 (4.5%)	0.005
*Other supporting adult only*	2 (1.2%)	2 (3%)	
*Multiple supporting adults*	31 (18%)	4 (6%)	
Co-Parent/FOB FC Attendance *	116 (69%)	36 (54%)	0.039
Grandparent FC Attendance *	39 (23%)	7 (10%)	0.041

Abbreviations: FOB, father of the baby; MRI, magnetic resonance imaging; Echo, echocardiogram; NS, not significant; ^#^ data presented as n (%), mean ± SD; * *p* < 0.05.

**Table 4:** Utilization of fetal–neonatal center services stratified by ADI deciles.

Cohort Characteristics ^#^	Low ADI (n = 135)	High ADI (n = 91)	*p* Value
MFM Consultation	135 (100%)	91 (100%)	NS
Fetal Imaging			
*Level 2 ultrasound*	135 (100%)	91 (100%)	NS
*Fetal MRI*	34 (25%)	31 (34%)	NS
Fetal echo *	87 (64%)	57 (63%)	NS
Genetic Counseling Visit	103 (76%)	65 (71%)	NS
FC Meeting Utilization			
Offered *	114 (84%)	81 (91%)	NS
Accepted *	108 (80%)	75 (82%)	NS
Attended *	103 (76%)	73 (80%)	NS
FC Participants			
Total number attending *	2.07 ± 0.83	1.88 ± 0.95	0.039
FC Supporting-Adult Composition *			
*No support person*	16 (15%)	26 (36%)	
*Co-parent or partner only*	66 (63%)	29 (40%)	
*Grandparent only*	6 (6%)	7 (10%)	0.012
*Other supporting adult only*	2 (2%)	1 (1%)	
*Multiple supporting adults*	14 (13%)	10 (14%)	
Co-Parent/FOB FC Attendance *	78 (75%)	38 (52%)	0.003
Grandparent FC Attendance *	16 (15%)	16 (22%)	NS

Abbreviations: FOB, father of the baby; MRI, magnetic resonance imaging; Echo, echocardiogram; NS, not significant; ^#^ data presented as n (%) or mean ± SD; * *p* < 0.05.

## Data Availability

The data presented in this study are available upon request from the corresponding author (with data availability limited by patient privacy).

## Abbreviations

The following abbreviations are used in this manuscript:

SDOH	Social Drivers of Health
FC	Family Complex Care Coordination Conference
ADI	Area Deprivation Index
FNMC	Fetal and Neonatal Medicine Center
MFM	Maternal–Fetal Medicine
NICU	Neonatal Intensive Care Unit
QI	Quality Improvement
IRB	Institutional Review Board
IUFD	Intrauterine Fetal Demise
LOS	Length of Stay
